# Global trends in typhoid and paratyphoid, and invasive non-typhoidal *salmonella*, and the burden of antimicrobial resistance: a trend analysis study from 1990 to 2021

**DOI:** 10.3389/fmed.2025.1588507

**Published:** 2025-05-20

**Authors:** Ke Shi, Tongdeng You

**Affiliations:** Department of Clinical Laboratory, Fuzhou Changle District People’s Hospital, Fuzhou, Fujian, China

**Keywords:** typhoid and paratyphoid, Invasive Non-typhoidal *Salmonella*, antimicrobial resistance, incidence, DALYs, mortality

## Abstract

**Objectives:**

To assess the global burden of typhoid and paratyphoid fever, and Invasive Non-typhoidal *Salmonella* (iNTS) from 1990 to 2021, and explore the burden of antimicrobial resistance (AMR) in *Salmonella*.

**Methods:**

Data were sourced from the Global Burden of Disease Study (GBD) 2021, focusing on age–standardized incidence rate (ASIR), mortality rate (ASMR), disability–adjusted life years rate (ASDR), and annualized percent change (EAPC).

**Results:**

From 1990 to 2021, the global burden of typhoid and paratyphoid fever decreased (EAPC = −4.15; 95% CI: −4.45 to −3.85). In contrast, the burden of iNTS showed a slow increasing trend (EAPC = 0.45; 95% CI: −0.32 to 1.22). The major epidemic trends were concentrated in regions with low and middle Socio–demographic Index (SDI). In high SDI regions, the age group most affected by deaths was 75 years and older, whereas in low SDI regions, particularly for typhoid and paratyphoid fever, deaths were more prevalent among children aged 0–14 years. Disability–adjusted life years (DALYs) and deaths due to multidrug–resistant *Salmonella* have decreased annually.

**Conclusion:**

While the global burden of typhoid and paratyphoid fever has declined, the burden of iNTS continues to rise slowly. The growing antimicrobial resistance of Salmonella further exacerbates the global disease burden.

## 1 Introduction

Salmonellosis is a common foodborne gastrointestinal infection, encompassing both invasive *Salmonella* infections (Typhoid and Paratyphoid) and Invasive Non-typhoidal *Salmonella* (iNTS). The disease typically manifests with acute symptoms such as fever, chills, and abdominal pain; however, severe infections can lead to systemic bloodstream infections, septic shock, and even death (1). *Salmonella* primarily invades the host’s intestinal epithelial cells through contaminated food or water, causing inflammation and electrolyte imbalances. The secreted endotoxins and exotoxins further exacerbate the intestinal inflammatory response and cause secretory diarrhea. It is estimated that in 2017, there were 143,000 cases of typhoid and paratyphoid fever globally, resulting in 135,900 deaths, along with 535,000 cases of non–typhoidal invasive *Salmonella* disease, which caused 77,500 deaths (2, 3).

According to the 2021 Global Burden of Disease (GBD) data, regions such as Africa and South Asia, particularly India, bear the heaviest burden of *Salmonella* (4, 5). In 2022, the European Union reported 65,208 confirmed cases of human salmonellosis, with 38.9% of cases requiring hospitalization and a mortality rate of 0.22% (6). In the United States, *Salmonella* accounted for 20% of foodborne disease outbreaks (7). In Central Africa, *Salmonella* species were the leading cause of diarrheal infections, accounting for 55.56% of cases (8). The transmission and mortality of *Salmonella* are closely linked to factors such as poor environmental sanitation, inadequate personal hygiene, malnutrition, and HIV infection (9, 10).

Furthermore, numerous studies have highlighted the widespread increase in antibiotic resistance of *Salmonella* in endemic regions, especially to fluoroquinolones (11). Some studies have shown that high levels of ciprofloxacin resistance in South Asia reach 20%. In 2020, extensively drug–resistant (XDR) typhoid fever accounted for 70% of cases in Pakistan, while ciprofloxacin resistance in South Africa exceeded 85% (12). In the European Union, resistance to ampicillin (29.8%), sulfonamides (30.1%), and tetracyclines (31.2%) was generally found to be at a high level among *Salmonella* isolates from human infections (13). Furthermore, due to factors such as international travel, cross–border trade, and geographical spread, imported *Salmonella* infections have led to an increase in multidrug–resistant strains in non–endemic areas (14). Therefore, understanding the global epidemiological trends of *Salmonella* and the antimicrobial resistance (AMR) status in various regions is crucial for developing effective control and prevention strategies.

This study utilizes data from the Global Burden of Disease (GBD) 1990–2021 to analyze the epidemiological trends of Salmonella infections and their changes over time, with the aim of better understanding this increasingly severe global public health issue. Additionally, we analyzed the global antimicrobial resistance burden of Salmonella infections from 1990 to 2021.

## 2 Materials and methods

### 2.1 Data source

This study adheres to the GATHER checklist^1^ and utilizes the Global Burden of Disease (GBD) study data released in 2024. It evaluates the incidence, mortality, and disability adjusted life year (DALYs) for 371 diseases and 811 regions across 204 countries and territories from 1990 to 2021 (4). Specifically, we extracted data on the incidence, mortality, and DALYs for Typhoid and Paratyphoid, and Invasive Non-typhoidal *Salmonella* (iNTS) from the Global Health Data Exchange (GHDx) query tool for the period 1990–2021, and further analyzed these data by age, sex, country, and region^2^.

We employed age–standardized rates (ASR) per 100,000 population, including age–standardized incidence rate (ASIR), age–standardized mortality rate (ASMR), and age–standardized DALYs rate (ASDR), to analyze data across all age groups. Disease modeling was conducted based on the collected and analyzed data. The epidemiological estimation methods used in GBD have been described in detail in other published literature (4, 15). This study was approved by the University of Washington in Seattle, WA, which waived informed consent because only de–identified and aggregated data were utilized.

### 2.2 Model building

The GBD 2019 uses the DisMod–MR 2.1 meta–regression tool, which is based on a Bayesian model framework, to model epidemiology. Using mathematical modeling, it estimates indicators such as disease incidence, mortality, disease progression, and healthy life expectancy from both mortality and non–fatal data. The modeling process is as follows: (1). Data aggregation and adjustment. (2). Data consistency check. (3). Integrated modeling and estimation. (4). Global estimates and application. (5). Model validation and optimization. (6). Results interpretation and application. DALYs are a summary measure of overall health loss, calculated by summing the years of life lost (YLL) and years lived with disability (YLD) for each cause of death (16).

### 2.3 Statistical analysis

In this study, age–standardized rates (ASR) were used to describe the incidence, DALYs, and mortality rates. All estimated rates are presented per 100,000 population. The uncertainty intervals (UI) are represented by the 2.5th and 97.5th percentiles, which indicate the range of the results. To estimate the time trends of Typhoid and Paratyphoid, and Invasive Non-typhoidal *Salmonella* (iNTS), we used the annual percent change (EAPC). EAPC values and their 95% confidence intervals (CI) were calculated using linear regression models. The method for determining trends is as follows: If EAPC > 0, it indicates that the variable is increasing (rising) over time; if EAPC < 0, it indicates that the variable is decreasing (falling) over time (17). The Sociodemographic Index (SDI) is an index that comprehensively assesses the development level of regions based on socioeconomic indicators. SDI typically considers multiple factors, such as population density, education level, life expectancy, and economic status, with values ranging from 0 to 1. Lower SDI values represent lower socioeconomic development levels, while higher values indicate higher development levels (18). All statistical analyses were conducted using R software (version 4.4.2).

## 3 Results

### 3.1 Global trends in *Salmonella* infections

According to data from 1990 to 2021, the age–standardized incidence rates (ASR) of typhoid and paratyphoid fever and Invasive Non-typhoidal *Salmonella* (iNTS) show markedly different trends. In 2021, the global age–standardized incidence rate for typhoid and paratyphoid fever was 127.77 (99.50–163.19), compared to 461.34 (370.05–574.72) in 1990, with an Estimated Annual Percent Change (EAPC) of −4.15 (−4.45 to −3.85), indicating a significant downward trend. In contrast, the global age–standardized incidence rate for Invasive Non-typhoidal *Salmonella* in 2021 was 7.21 (5.83–8.64), compared to 5.986 (4.937–7.034) in 1990, with an EAPC of 0.45 (−0.32 to 1.22), reflecting an upward trend (Table 1).

**TABLE 1:** ASIR, ASDR, and ASMR rates of typhoid and paratyphoid, and Invasive Non-typhoidal *Salmonella* (iNTS) by SDI and region in 2021.

Categories	ASIR (95% UI)		ASDR (95% UI)		ASMR (95% UI)	
	Typhoid and paratyphoid	Invasive Non-typhoidal *Salmonella* (iNTS)	Typhoid and paratyphoid	Invasive Non-typhoidal *Salmonella* (iNTS)	Typhoid and paratyphoid	Invasive Non-typhoidal *Salmonella* (iNTS)
Global	127.77 (99.50–163.19)	7.21 (5.83–8.64)	115.26 (59.32–198.47)	69.14 (39.70–111.21)	1.50 (0.78–2.54)	0.88 (0.52–1.41)
High SDI	1.54 (1.28–1.88)	0.89 (0.67–1.13)	0.56 (0.22–1.25)	0.63 (0.39–0.99)	0.01 (0.00–0.02)	0.01 (0.01–0.02)
High–middle SDI	20.49 (15.99–26.09)	0.67 (0.51–0.85)	13.05 (6.66–21.60)	1.61 (0.85–2.83)	0.17 (0.09–0.28)	0.03 (0.02–0.05)
Low SDI	170.98 (133.61–219.66)	20.91 (17.08–24.83)	155.55 (78.33–278.95)	234.06 (135.29–376.15)	2.15 (1.07–3.77)	3.30 (1.91–5.19)
Low–middle SDI	258.10 (203.11–327.54)	7.47 (6.13–8.84)	219.50 (114.29–364.65)	49.60 (28.70–76.60)	2.96 (1.55–4.88)	0.71 (0.41–1.09)
Middle SDI	85.37 (66.93–108.83)	2.72 (2.21–3.30)	66.38 (34.63–111.64)	11.81 (6.41–19.46)	0.89 (0.46–1.48)	0.17 (0.09–0.27)
Andean Latin America	1.39 (1.01–1.93)	0.67 (0.45–0.92)	0.41 (0.22–0.72)	2.19 (1.13–3.89)	0.01 (0.01–0.02)	0.04 (0.02–0.07)
Australasia	0.19 (0.11–0.29)	0.42 (0.25–0.62)	0.00 (0.00–0.00)	0.15 (0.12–0.20)	0.00 (0.00–0.00)	0.01 (0.00–0.01)
Caribbean	9.18 (7.38–11.42)	0.47 (0.30–0.66)	8.80 (4.10–17.63)	1.13 (0.51–2.02)	0.11 (0.05–0.22)	0.02 (0.01–0.03)
Central Asia	0.38 (0.29–0.50)	0.43 (0.28–0.62)	0.09 (0.06–0.15)	0.40 (0.20–0.73)	0.00 (0.00–0.00)	0.01 (0.00–0.01)
Central Europe	0.35 (0.26–0.47)	0.41 (0.26–0.61)	0.02 (0.01–0.02)	0.29 (0.19–0.43)	0.00 (0.00–0.00)	0.01 (0.01–0.01)
Central Latin America	13.14 (10.35–17.14)	0.70 (0.51–0.90)	1.39 (1.17–1.67)	0.37 (0.21–0.63)	0.03 (0.03–0.04)	0.01 (0.00–0.01)
Central Sub–Saharan Africa	23.51 (17.82–30.35)	28.34 (23.29–33.21)	17.77 (8.43–34.48)	198.36 (107.65–312.48)	0.25 (0.12–0.49)	3.50 (1.92–5.48)
East Asia	9.30 (7.44–11.68)	0.52 (0.34–0.74)	4.95 (2.28–9.40)	1.79 (0.89–3.30)	0.07 (0.03–0.13)	0.04 (0.02–0.06)
Eastern Europe	0.39 (0.31–0.50)	0.46 (0.28–0.67)	0.03 (0.02–0.05)	0.50 (0.30–0.82)	0.00 (0.00–0.00)	0.01 (0.01–0.02)
Eastern Sub–Saharan Africa	117.84 (91.25–149.71)	8.28 (6.96–9.64)	131.05 (63.23–234.90)	55.97 (32.72–86.65)	1.84 (0.90–3.25)	0.83 (0.48–1.29)
High–income Asia Pacific	0.27 (0.19–0.37)	0.54 (0.30–0.80)	0.01 (0.01–0.02)	0.17 (0.07–0.35)	0.00 (0.00–0.00)	0.00 (0.00–0.01)
High–income North America	0.42 (0.31–0.56)	0.94 (0.65–1.27)	0.03 (0.03–0.04)	0.25 (0.22–0.29)	0.00 (0.00–0.00)	0.01 (0.01–0.01)
North Africa and Middle East	19.16 (14.86–25.13)	1.82 (1.41–2.28)	15.28 (7.42–27.73)	10.33 (5.60–17.13)	0.20 (0.10–0.37)	0.17 (0.09–0.28)
Oceania	293.05 (222.33–391.94)	1.91 (1.45–2.42)	279.22 (134.43–513.01)	15.25 (8.17–25.08)	3.81 (1.81–6.97)	0.26 (0.14–0.42)
South Asia	379.64 (295.92–484.02)	3.42 (2.66–4.17)	311.91 (164.59–516.19)	23.65 (13.17–38.20)	4.15 (2.18–6.87)	0.37 (0.21–0.59)
Southeast Asia	151.55 (117.82–194.66)	2.60 (2.01–3.20)	138.10 (66.93–235.33)	12.66 (6.97–20.75)	1.86 (0.90–3.13)	0.23 (0.12–0.37)
Southern Latin America	0.66 (0.52–0.82)	0.57 (0.37–0.78)	0.04 (0.03–0.05)	0.02 (0.02–0.02)	0.00 (0.00–0.00)	0.00 (0.00–0.00)
Southern Sub–Saharan Africa	1.44 (1.10–1.84)	11.24 (8.49–15.80)	1.16 (0.52–2.19)	45.94 (23.24–76.53)	0.02 (0.01–0.03)	0.60 (0.31–1.01)
Tropical Latin America	1.15 (0.87–1.55)	0.55 (0.38–0.76)	0.18 (0.09–0.33)	1.19 (0.63–2.04)	0.00 (0.00–0.01)	0.02 (0.01–0.03)
Western Europe	0.64 (0.47–0.89)	0.76 (0.54–1.03)	0.01 (0.01–0.02)	0.11 (0.09–0.12)	0.00 (0.00–0.00)	0.00 (0.00–0.01)
Western Sub–Saharan Africa	109.27 (85.71–140.09)	47.54 (38.53–56.66)	122.69 (57.95–235.03)	486.81 (266.57–789.85)	1.63 (0.79–3.07)	6.88 (3.91–10.95)

ASIR, age–standardized incidence rate; ASDR, age standardized DALYs rate; ASMR, age standardized mortality rate; SDI, socio–demographic index; UI, uncertainty interval.

In 2021, the global age–standardized DALYs for typhoid and paratyphoid fever was 115.26 (59.32–198.47), while in 1990 it was 301.362 (158.155–519.105), with an EAPC of −2.99 (−3.23 to −2.74), again showing a decreasing trend. This indicates that the health burden associated with typhoid and paratyphoid fever has decreased globally. Conversely, the global age–standardized DALYs for Invasive Non-typhoidal *Salmonella* (iNTS) in 2021 was 69.14 (39.70–111.21), compared to 61.696 (35.538−98.757) in 1990, with an EAPC of 0.52 (−0.23 to 1.27), reflecting an upward trend (Table 1).

**FIGURE 1:**
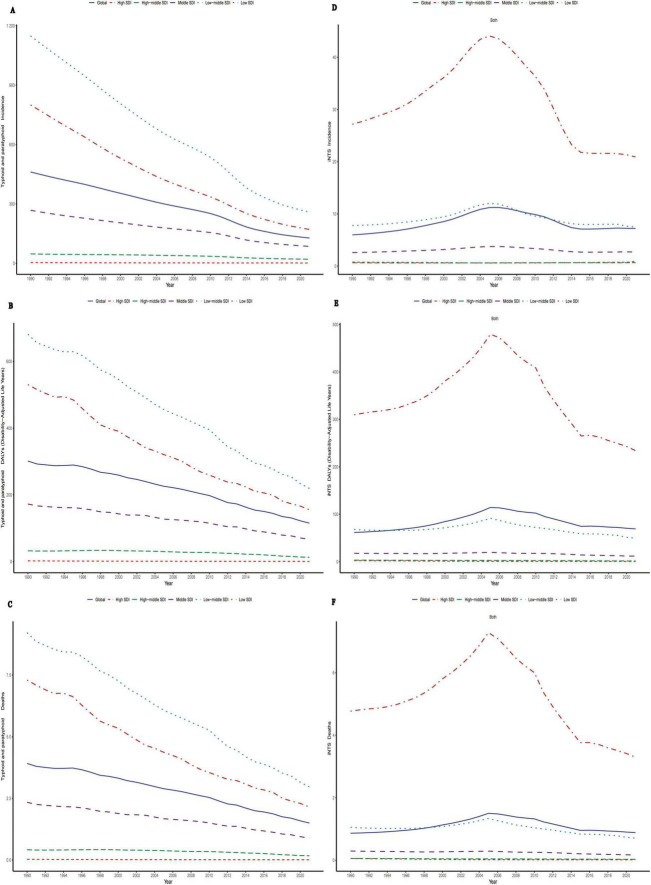
Temporal trends in age-standardized incidence (ASIR), DALYs (ASDR), and mortality rates (ASMR) for typhoid and paratyphoid, and invasive non-typhoidal *Salmonella* (iNTS) from 1990–2021 by global and SDI. **(A)** ASIR, **(B)** ASDR, and **(C)** ASMR of Typhoid and Paratyphoid. **(D)** ASIR, **(E)** ASDR, and **(F)** ASMR of iNTS. SDI, socio-demographic index.

The global age–standardized mortality rate for typhoid and paratyphoid fever in 2021 was 1.50 (0.78–2.54), compared to 3.916 (2.036–6.739) in 1990, with an EAPC of −2.70 (−2.85 to −2.55), indicating a downward trend in global mortality. In contrast, the global age–standardized mortality rate for Invasive Non-typhoidal *Salmonella* (iNTS) in 2021 was 0.88 (0.52–1.41), compared to 0.865 (0.500–1.357) in 1990, with an EAPC of 0.40 (−0.02 to 0.83), reflecting an upward trend. Overall, the incidence of typhoid and paratyphoid fever has decreased, while the incidence of Invasive Non-typhoidal *Salmonella* (iNTS) significantly increased between 1990 and 2005, then began to decline, though it remains on an upward trajectory overall (Figure 1).

### 3.2 Global trends by SDI and 21 regions

**FIGURE 2:**
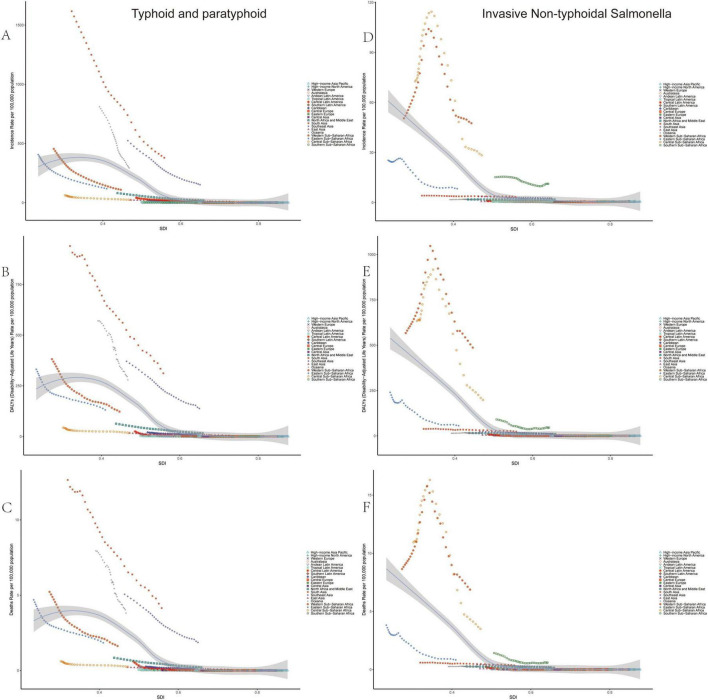
Temporal trends in age-standardized incidence (ASIR), DALYs (ASDR), and mortality rates (ASMR) for typhoid and paratyphoid, and invasive non-typhoidal *Salmonella* (iNTS) for 21 regions by SDI, 2009–2021. **(A)** ASIR, **(B)** ASDR, and **(C)** ASMR of typhoid and paratyphoid. **(D)** ASIR, **(E)** ASDR, and **(F)** ASMR of iNTS. SDI, socio-demographic index.

In 2021, the age–standardized incidence rates, DALYs, and mortality rates for typhoid and paratyphoid fever were highest in low and middle SDI regions, with values of 258.10 (203.11–327.54), 219.50 (114.29–364.65), and 2.96 (1.55–4.88), respectively. These indicators showed a concave downward trend as SDI increased, eventually approaching zero. In contrast, the age–standardized incidence rates, DALYs, and mortality rates for Invasive Non-typhoidal *Salmonella* (iNTS) were highest in low SDI regions, significantly exceeding those of other regions, with values of 20.91 (17.08–24.83), 234.06 (135.29–376.15), and 3.30 (1.91–5.19), respectively. As SDI increased, these indicators gradually decreased. Although the age–standardized incidence rate for iNTS was lower than that of typhoid and paratyphoid fever in low SDI regions, the DALYs and mortality rates for iNTS exceeded those of typhoid and paratyphoid fever, indicating that the burden of iNTS was more severe in these regions (Figure 2 and Table 1).

Regionally, the highest age–standardized incidence rates, DALYs, and mortality rates for typhoid and paratyphoid fever were found in South Asia, with values of 379.64 (295.92–484.02), 311.91 (164.59–516.19), and 4.15 (2.18–6.87), respectively. For iNTS, the highest age–standardized incidence rates, DALYs, and mortality rates were observed in Sub–Saharan Africa, with values of 47.54 (38.53–56.66), 486.81 (266.57–789.85), and 6.88 (3.91–10.95), respectively (Supplementary Tables 1, 2, and Figure 2).

Overall, both typhoid and paratyphoid fever and Invasive Non-typhoidal *Salmonella* showed a general downward trend from 1990 to 2021. However, the trends of these two diseases differ significantly, particularly in low– and middle–SDI regions. In high–SDI regions, the burden of both diseases is similar.

### 3.3 Global trends by country

In 2021, the highest age–standardized incidence rate for typhoid and paratyphoid fever was in India, at 411.46 (318.42–529.58), consistent with the high incidence rate in the South Asia region. In terms of DALYs and mortality rate, Bhutan showed the highest figures, with values of 463.24 (237.57–779.34) and 6.01 (3.08–10.07), and the country also had a relatively high age–standardized incidence rate.

For Invasive Non-typhoidal *Salmonella* (iNTS), the highest age–standardized incidence rate, DALYs, and mortality rate were found in Mali, with values of 76.24 (62.19–91.08), 859.23 (483.46–1357.04), and 13.26 (7.54–20.67), respectively.

Regarding growth rates, the United Kingdom had the highest annual percentage change (EAPC) in the age–standardized incidence rate for both typhoid and paratyphoid fever and Invasive Non–typhoidal *Salmonella*, with EAPCs of 1.27 (1.16–1.37) and 3.95 (3.37–4.53), respectively. In terms of DALYs EAPC growth, Czechia and the Bahamas ranked highest, with values of 7.50 (4.96–10.10) and 5.60 (3.75–7.48). As for mortality rate EAPC, Norway and Jamaica showed the fastest growth rates, with EAPCs of 5.65 (4.56–6.75) and 10.98 (8.85–13.16), respectively. Notably, although Norway and Jamaica had high EAPC growth for DALYs, the Bahamas showed a decreasing trend in the age–standardized incidence rate EAPC, while its DALYs and mortality rate EAPCs exhibited significant growth (Supplementary Tables 3, 4).

**FIGURE 3:**
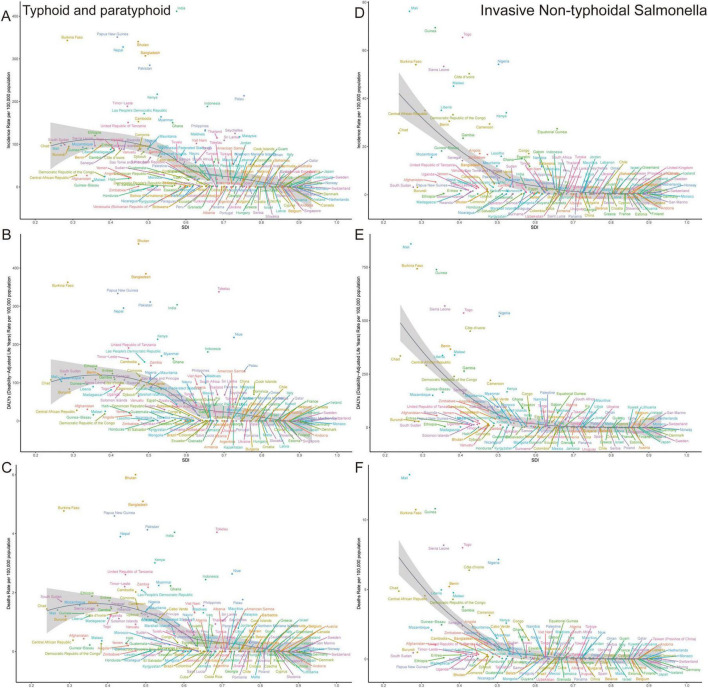
Age-standardized incidence (ASIR), DALYs (ASDR), and mortality rates (ASMR) for typhoid and paratyphoid, and invasive non-typhoidal *Salmonella* (iNTS) in 204 countries in 2021. **(A)** ASIR, **(B)** ASDR, and **(C)** ASMR of typhoid and paratyphoid. **(D)** ASIR, **(E)** ASDR, and **(F)** ASMR of iNTS.

**FIGURE 4:**
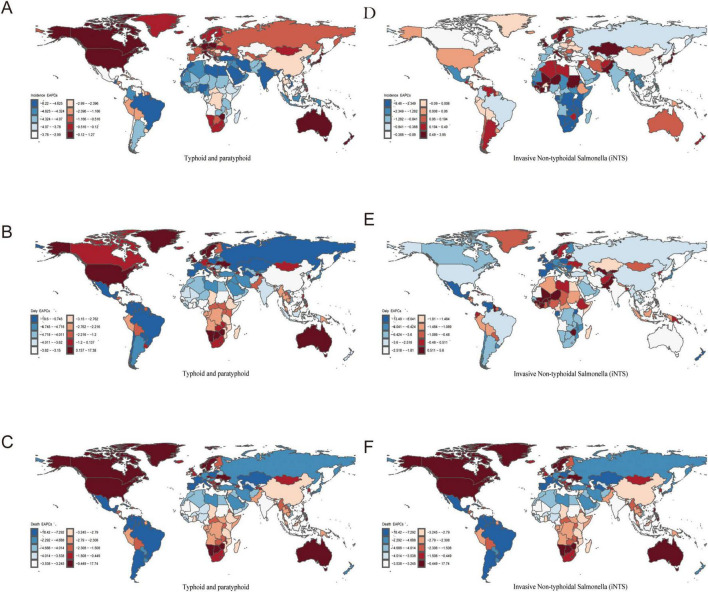
The EAPC for typhoid and paratyphoid, and invasive non-typhoidal *Salmonella* (iNTS) in 204 countries from 1990–2021. **(A)** ASIR, **(B)** ASDR, and **(C)** ASMR of typhoid and paratyphoid. **(D)** ASIR, **(E)** ASDR, and **(F)** ASMR of iNTS. EAPC, estimated annual percentage changes. ASIR, age-standardized incidence rate; ASDR, age standardised DALYs rate; ASMR, age standardised mortality rate.

These trends highlight the substantial differences in the burden and growth rates of Typhoid, Paratyphoid, and Invasive Non-typhoidal *Salmonella* across countries worldwide (Figures 3, 4).

### 3.4 Age trends and differences

In the global trends of *Salmonella* infections, Typhoid and Paratyphoid, as well as iNTS, have the highest incidence, DALYs, and mortality rates among children under 5 years old. These indicators gradually decrease as age increases, and males generally have higher incidence rates than females (Supplementary Figure 1).

From 1990 to 2021, the age distribution of *Salmonella* infections has not significantly changed in most regions, except for differences observed in Australasia and Western Europe. In terms of age–standardized incidence rates, typhoid and paratyphoid are predominantly seen in the over–75 age group in Tropical Latin America, Southern Latin America, High–income North America, High–income Asia Pacific, Central Latin America, Central Europe, and Andean Latin America regions, with incidence rates exceeding 50% in these areas. In contrast, the incidence of Invasive Non-typhoidal *Salmonella* (iNTS) is mainly concentrated in the 0–14 age group.

Regarding DALYs, except in Western Europe, Central Europe, and High–income North America, the DALYs for the over–75 age group exceed 49%, while the 0–14 age group still dominates in most other regions. The DALYs trend for iNTS mirrors the incidence rate, with a predominant concentration in the 0–14 age group.

**FIGURE 5:**
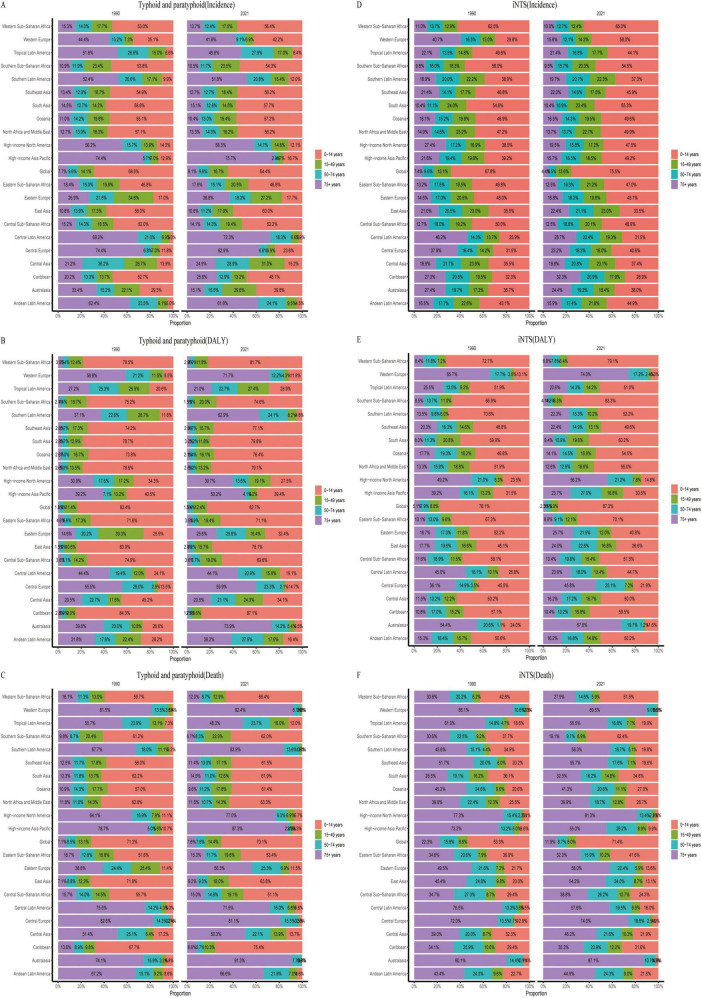
Age composition ratios of age-standardized incidence (ASIR), DALYs (ASDR), and mortality rates (ASMR) for typhoid and paratyphoid, and invasive non-typhoidal *Salmonella* (iNTS) in 2009–2021. **(A)** ASIR, **(B)** ASDR, and **(C)** ASMR of typhoid and paratyphoid. **(D)** ASIR, **(E)** ASDR, and **(F)** ASMR of iNTS.

As for mortality rates, in economically developed regions such as Western Europe, High–income North America, High–income Asia Pacific, Central Europe, and Australasia, the mortality rate in the over–75 age group accounts for more than 70%. In economically underdeveloped regions, the mortality proportion is higher in the 0–14 age group. Overall, the mortality proportion for iNTS also tends to be higher in the over–75 age group in most regions (Figure 5).

### 3.5 Antimicrobial resistance burden

**FIGURE 6:**
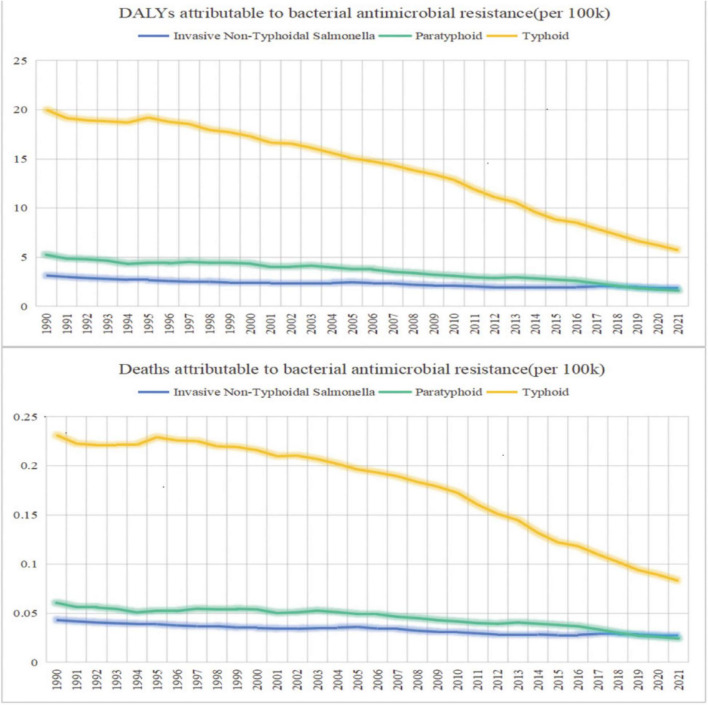
Trends in the DALYs and deaths attributable to bacterial antimicrobial resistance 1990–2021.

From 1990 to 2021, DALYs and deaths due to multidrug–resistant *Salmonella* have decreased annually. In 2021, the DALYs for Typhoid, Paratyphoid, and iNTS were 5.72 (0.62–10.81), 1.60 (0.23–2.98), and 1.83 (0.27–3.38), respectively; while deaths were 0.075 (0.0091–0.14), 0.023 (0.0036–0.041), and 0.025 (0.002–0.047) (Figure 6).

## 4 Discussion

*Salmonella* infections are a significant public health issue globally. This study analyzes the trends in age–standardized incidence rates, disease burden DALYs, and mortality rates for typhoid, paratyphoid, and iNTS from 1990 to 2021. It also explores the burden of AMR in *Salmonella*. The findings indicate that the incidence of typhoid and paratyphoid fever has been declining from 1990 to 2021, while the infection rate of iNTS has been slowly increasing. There are significant differences across regions and countries. For instance, South Asia has the highest incidence rates, disease burden, and mortality for typhoid and paratyphoid, while sub–Saharan Africa is a hotspot for iNTS infections. Another notable difference lies in economic and healthcare levels. Regions with a higher SDI tend to have lower incidence and mortality rates, with deaths primarily concentrated in the elderly population, whereas regions with lower SDI show higher infection rates, mostly affecting children.

This study found that the global incidence of typhoid and paratyphoid fever is declining, while iNTS infections are on the rise. Although the incidence of typhoid and paratyphoid fever is 17 times higher than that of iNTS, the disease burden DALYs and mortality rates for iNTS have surpassed those of typhoid and paratyphoid fever and continue to increase annually. This indicates that the harm caused by iNTS infections is more severe. Possible reasons for this trend include: (1). iNTS is a common source of infection in sub–Saharan Africa (19), a region with low economic and healthcare levels. (2). There is a lack of effective vaccines targeting iNTS (20). (3). The majority of serotype strains isolated currently are *Salmonella* Typhimurium and *Salmonella* Enteritidis, which account for over 90% of all iNTS serotypes. These strains possess more potent virulence genes and antibiotic resistance phenotypes (21, 22).

Furthermore, we observed regional differences in the prevalence of typhoid, paratyphoid fever, and iNTS. Typhoid and paratyphoid fever are most prevalent in South Asia, particularly in India, while iNTS infections are primarily endemic in countries like Mali in Africa. However, some studies from India indicate that iNTS has also become a significant public health issue there, posing a severe threat to population health (23). Despite the Indian government’s efforts to enhance regulations on water, environmental sanitation, and food safety, the situation is aggravated by hot climate conditions (24), and long–term antibiotic use, which has led to increased antibiotic resistance in *Salmonella*, further exacerbating India’s public health burden (5, 25).

On the other hand, globalization and rising temperatures have increased the risk of imported *Salmonella* infections. A study from China revealed that over 90% of imported *Salmonella* cases came from Indonesia, India, Myanmar, and Cambodia (26). Notably, *Salmonella* infections have been rising in the United Kingdom and have become the second–largest zoonotic pathogen in the European Union (6). A European Union survey revealed that, among 4,135 travel–associated cases, 77.8% involved regions outside the EU (6). In fact, from September to November 2023, an outbreak of *Salmonella* infections occurred in the United Kingdom (27). Therefore, *Salmonella* infections have become a global burden, and we must remain vigilant about the health risks posed by imported cases.

The study also found significant differences in the distribution of incidence rates, disease burden DALYs, and mortality rates of typhoid, paratyphoid, and iNTS across different age groups, which varies by region. In the Americas, the incidence of typhoid and paratyphoid fever is higher, particularly among those aged 75 and above. In contrast, iNTS primarily affects children aged 0–14 years. Moreover, the study revealed substantial disparities in mortality rates due to *Salmonella* infections. Typhoid and paratyphoid fever mainly affect elderly populations aged 75 and above in economically developed regions, such as Western Europe, North America, high–income Asia–Pacific regions, Central Europe, and Oceania, where they account for over 70% of deaths. In less economically developed areas, children aged 0–14 years are more commonly affected, a finding supported by some studies (11, 28).

On the other hand, iNTS infections pose a major threat to individuals aged 75 and above in most regions. This increased vulnerability may be attributed to physiological factors such as the decline of the immune system in the elderly, which makes them more susceptible to iNTS infections. Although developed countries typically have superior healthcare systems, higher living standards, good nutrition, and sound public health policies—factors that contribute to longer life expectancies—these countries also face the challenges of an aging population, which in turn exacerbates the health burden in these regions. Some studies have also indicated that elderly individuals are more likely to become long–term carriers of *Salmonella*, exacerbating their health burdens (29). In contrast, in economically underdeveloped regions, children aged 0–14 years bear the brunt of infection, primarily due to limited healthcare resources, malnutrition, and other infectious diseases, all of which adversely impact children’s health and development (30).

In recent years, due to the misuse of antibiotics or insufficient regulation, the prevalence of MDR *Salmonella* has been rising (31, 32). In the United States, *Salmonella* carrying the extended–spectrum beta–lactamase gene (*blaCTX–M–65*) has been widely spread (33). These resistant strains pose an increased risk of bloodstream infections, thereby exacerbating the health burden on patients, especially in endemic regions (34, 35).

Some studies have shown that the main mechanism of fluoroquinolone resistance in *Salmonella* is mediated by QRDR and PMQR genes (36), with plasmids playing a key role in the development of resistance (37). Furthermore, many resistance genes are often located on plasmids, such as fluoroquinolone resistance genes (*qnrA, qnrB*) and β–lactam resistance genes (*blaTEM, blaSHV, blaCTX–M, blaCMY–2*) (38). Plasmids, as mobile genetic elements (MGE), are the primary reservoir for antimicrobial resistance genes (39). With plasmid transfer, they can spread between different bacterial genera, facilitating the development of resistance to multiple antibiotics, significantly increasing the difficulty of infection treatment.

In summary, while the overall burden of *Salmonella* infections is gradually decreasing compared to the past, the increasing prevalence of MDR strains due to antibiotic misuse and insufficient regulation has intensified the global burden of *Salmonella* infections. Addressing this issue requires not only the prudent use of antibiotics but also improvements in sanitation, healthcare, and accelerated vaccine development to reduce the global impact of *Salmonella* infections.

## 5 Limitations

The study has several limitations. First, the GBD data may either underestimate or overestimate the burden of certain diseases, particularly in low– and middle–income countries, where health reporting systems are often weaker, leading to incomplete or inaccurate data. Second, inconsistencies in the data may introduce biases in cross–country comparisons, which could affect the analysis of policies or interventions in different nations. Finally, many of the data in the GBD database are derived from model estimates, particularly in the absence of direct observational data. While models help fill data gaps, their predictive results may contain uncertainties.

## Data Availability

The original contributions presented in the study are included in the article/Supplementary material, further inquiries can be directed to the corresponding author.

## Footnotes

1
https://www.who.int/publications/m/item/gather-checklist

2
https://vizhub.healthdata.org/gbd-results/

## Supplementary material

Supplementary Figure 1 Difference in age specific of ASIR, ASDR, and ASMR rates between men and women in 2021. ASIR, age–standardized incidence rate; ASDR, age standardized DALYs rate; ASMR, age standardized mortality rate.
