# Supplementary material for: Global trends in typhoid and paratyphoid, and invasive non-typhoidal salmonella, and the burden of antimicrobial resistance: a trend analysis study from 1990 to 2021
Source: Front Med (Lausanne). 2025 May 20;12:1588507. doi: 10.3389/fmed.2025.1588507 (PMC12129787; doi:10.3389/fmed.2025.1588507)
Supplement: Supplementary file 1 [file Table_1.docx]

| **Table S1 ASIR , ASDR , ASMR Rates of Typhoid and paratyphoid, and Invasive Non-typhoidal Salmonella (iNTS) by SDI and Region in 1990** | | | | | | |
| --- | --- | --- | --- | --- | --- | --- |
| **Categories** | **ASIR (95% UI)** | | **ASDR (95% UI)** | | **ASMR (95% UI)** | |
|  | Typhoid and paratyphoid | Invasive Non-typhoidal Salmonella (iNTS) | Typhoid and paratyphoid | Invasive Non-typhoidal Salmonella (iNTS) | Typhoid and paratyphoid | Invasive Non-typhoidal Salmonella (iNTS) |
| Global | 461.341(370.055-574.723) | 5.986(4.937-7.034) | 301.362(158.155-519.105) | 61.696(35.538-98.757) | 3.916(2.036-6.739) | 0.865(0.500-1.357) |
| High SDI | 3.894(3.031-5.055) | 0.761(0.601-0.941) | 2.251(0.818-5.023) | 2.386(1.761-3.272) | 0.028(0.011-0.063) | 0.054(0.044-0.068) |
| High-middle SDI | 47.537(37.394-60.472) | 0.642(0.499-0.798) | 32.393(16.102-56.549) | 3.371(1.930-5.479) | 0.415(0.207-0.712) | 0.057(0.035-0.090) |
| Low SDI | 799.618(629.765-1015.325) | 27.169(22.613-31.572) | 530.304(257.579-949.376) | 309.464(173.743-503.054) | 7.297(3.540-12.759) | 4.768(2.667-7.425) |
| Low-middle SDI | 1148.728(911.117-1432.769) | 7.779(6.477-9.104) | 681.212(358.020-1166.771) | 68.040(39.255-104.029) | 9.217(4.751-15.679) | 1.053(0.600-1.604) |
| Middle SDI | 267.905(214.048-331.228) | 2.595(2.131-3.192) | 172.623(89.977-286.359) | 17.681(10.580-26.626) | 2.338(1.228-3.825) | 0.295(0.180-0.428) |
| Andean Latin America | 2.735(2.033-3.785) | 0.660(0.444-0.903) | 12.835(11.335-14.284) | 3.195(1.750-5.159) | 0.273(0.238-0.308) | 0.057(0.029-0.093) |
| Australasia | 0.147(0.087-0.230) | 0.371(0.227-0.539) | 0.006(0.005-0.008) | 0.440(0.377-0.521) | 0.000(0.000-0.000) | 0.013(0.011-0.015) |
| Caribbean | 19.813(15.949-24.679) | 0.464(0.318-0.654) | 20.217(9.618-38.972) | 1.073(0.511-1.970) | 0.264(0.130-0.496) | 0.018(0.009-0.032) |
| Central Asia | 1.082(0.842-1.347) | 0.421(0.269-0.607) | 0.889(0.600-1.257) | 0.581(0.332-0.925) | 0.017(0.012-0.024) | 0.009(0.005-0.015) |
| Central Europe | 0.411(0.321-0.531) | 0.412(0.266-0.581) | 0.043(0.035-0.053) | 2.570(2.242-2.938) | 0.002(0.001-0.002) | 0.052(0.045-0.059) |
| Central Latin America | 40.939(33.516-51.692) | 1.065(0.822-1.331) | 24.622(22.699-26.641) | 9.755(8.887-10.732) | 0.611(0.555-0.661) | 0.246(0.227-0.267) |
| Central Sub-Saharan Africa | 62.339(47.969-82.450) | 72.671(60.746-84.617) | 43.504(20.060-82.474) | 633.545(355.040-1022.844) | 0.607(0.277-1.103) | 10.940(6.044-17.525) |
| East Asia | 22.786(17.662-29.071) | 0.592(0.399-0.834) | 16.257(7.107-31.164) | 4.478(2.362-7.628) | 0.210(0.092-0.398) | 0.082(0.044-0.137) |
| Eastern Europe | 0.501(0.377-0.651) | 0.603(0.381-0.880) | 0.127(0.092-0.164) | 0.950(0.647-1.410) | 0.002(0.002-0.003) | 0.017(0.011-0.025) |
| Eastern Sub-Saharan Africa | 405.914(312.945-524.913) | 25.269(21.424-29.476) | 331.438(158.778-594.152) | 239.950(133.553-389.024) | 4.690(2.263-8.213) | 3.818(2.146-5.997) |
| High-income Asia Pacific | 0.292(0.220-0.385) | 0.334(0.181-0.506) | 0.036(0.028-0.049) | 0.462(0.270-0.793) | 0.001(0.001-0.001) | 0.011(0.007-0.016) |
| High-income North America | 0.410(0.307-0.537) | 0.640(0.468-0.812) | 0.023(0.020-0.025) | 0.882(0.821-0.954) | 0.000(0.000-0.000) | 0.024(0.022-0.027) |
| North Africa and Middle East | 81.338(63.636-105.965) | 1.856(1.424-2.342) | 63.194(29.526-116.437) | 16.155(8.861-25.348) | 0.848(0.397-1.543) | 0.275(0.152-0.424) |
| Oceania | 810.479(610.728-1040.629) | 1.693(1.303-2.105) | 570.541(263.282-1035.770) | 14.347(7.709-24.085) | 7.943(3.598-14.214) | 0.262(0.141-0.429) |
| South Asia | 1616.925(1282.409-2017.517) | 4.142(3.262-5.068) | 939.994(495.541-1585.664) | 38.506(20.540-62.358) | 12.650(6.550-20.929) | 0.598(0.325-0.950) |
| Southeast Asia | 523.636(409.776-654.664) | 3.579(2.815-4.295) | 370.855(185.638-648.917) | 23.865(13.322-37.205) | 5.059(2.508-8.812) | 0.430(0.244-0.655) |
| Southern Latin America | 2.182(1.676-2.726) | 0.490(0.339-0.677) | 3.144(2.799-3.503) | 0.100(0.086-0.117) | 0.074(0.065-0.084) | 0.001(0.001-0.002) |
| Southern Sub-Saharan Africa | 1.688(1.267-2.180) | 15.179(11.320-24.073) | 1.113(0.518-2.149) | 90.664(45.960-174.232) | 0.015(0.007-0.029) | 1.422(0.767-2.506) |
| Tropical Latin America | 6.295(4.802-8.361) | 0.664(0.475-0.859) | 2.210(1.351-3.577) | 3.788(2.694-5.344) | 0.047(0.029-0.076) | 0.069(0.050-0.093) |
| Western Europe | 0.630(0.471-0.873) | 0.459(0.312-0.630) | 0.076(0.066-0.086) | 1.118(1.042-1.212) | 0.002(0.002-0.003) | 0.041(0.037-0.045) |
| Western Sub-Saharan Africa | 454.061(349.025-586.272) | 50.475(41.208-59.457) | 381.509(176.996-678.251) | 566.788(320.476-918.777) | 5.220(2.482-9.155) | 8.644(4.887-13.465) |
| ASIR:age-standardized incidence rate; ASDR:Age standardised DALY rate; ASMR:Age standardised mortality rate; SDI, socio-demographic index;UI, uncertainty interval. | | | | | | |
