# Supplementary material for: Global trends in typhoid and paratyphoid, and invasive non-typhoidal salmonella, and the burden of antimicrobial resistance: a trend analysis study from 1990 to 2021
Source: Front Med (Lausanne). 2025 May 20;12:1588507. doi: 10.3389/fmed.2025.1588507 (PMC12129787; doi:10.3389/fmed.2025.1588507)
Supplement: Supplementary file 2 [file Table_2.docx]

| **Table S2 Trends in EAPC of Typhoid and Paratyphoid, and Invasive Non-Typhoidal Salmonella (iNTS) from 1990 to 2021** | | | | | | |
| --- | --- | --- | --- | --- | --- | --- |
| Location | **ASIR (95% CI)** | | **ASDR (95% CI)** | | **ASMR (95% CI)** | |
|  | Typhoid and paratyphoid | Invasive Non-typhoidal Salmonella (iNTS) | Typhoid and paratyphoid | Invasive Non-typhoidal Salmonella (iNTS) | Typhoid and paratyphoid | Invasive Non-typhoidal Salmonella (iNTS) |
| Global | -4.15(-4.45--3.85) | 0.45(-0.32-1.22) | -2.99(-3.23--2.74) | 0.52(-0.23-1.27) | -2.70(-2.85--2.55) | 0.40(-0.02-0.83) |
| High SDI | -2.65(-2.88--2.43) | -0.01(-0.37-0.36) | -4.04(-4.22--3.86) | -4.10(-4.65--3.55) | -3.81(-3.94--3.67) | -4.80(-5.20--4.41) |
| High-middle SDI | -2.66(-3.02--2.30) | 0.24(0.16-0.32) | -2.56(-3.07--2.04) | -2.13(-2.28--1.98) | -1.95(-2.26--1.64) | -1.88(-1.95--1.82) |
| Low SDI | -4.95(-5.12--4.79) | -1.16(-2.04--0.28) | -3.88(-4.00--3.77) | -0.84(-1.60--0.08) | -3.65(-3.74--3.57) | -0.81(-1.28--0.34) |
| Low-middle SDI | -4.82(-5.08--4.55) | -0.13(-0.70-0.45) | -3.54(-3.75--3.33) | -0.68(-1.20--0.16) | -3.30(-3.43--3.17) | -0.93(-1.22--0.64) |
| Middle SDI | -3.61(-3.86--3.35) | -0.03(-0.52-0.46) | -2.80(-3.04--2.56) | -1.07(-1.46--0.69) | -2.60(-2.74--2.46) | -1.50(-1.69--1.31) |
| Andean Latin America | -2.27(-2.37--2.16) | -0.06(-0.12--0.01) | -11.37(-12.15--10.59) | -1.22(-1.34--1.09) | -10.11(-10.70--9.52) | -1.13(-1.21--1.06) |
| Australasia | 0.99(0.69-1.28) | 0.19(-0.04-0.42) | -0.10(-1.76-1.59) | -2.19(-4.70-0.39) | -0.03(-1.33-1.27) | -3.43(-5.00--1.83) |
| Caribbean | -2.37(-2.40--2.34) | -0.08(-0.19-0.02) | -2.32(-2.45--2.20) | 0.27(0.17-0.36) | -2.39(-2.50--2.27) | 0.09(0.03-0.15) |
| Central Asia | -3.22(-3.34--3.11) | -0.00(-0.15-0.14) | -7.75(-8.18--7.31) | -1.36(-1.53--1.18) | -7.08(-7.51--6.65) | -1.16(-1.26--1.06) |
| Central Europe | -0.24(-0.54-0.06) | -0.12(-0.41-0.17) | -3.63(-4.95--2.29) | -6.19(-7.46--4.90) | -5.26(-6.23--4.28) | -7.24(-8.15--6.32) |
| Central Latin America | -3.63(-3.91--3.35) | -1.31(-1.98--0.63) | -8.64(-9.55--7.72) | -9.63(-11.64--7.58) | -10.47(-11.12--9.82) | -12.43(-13.76--11.07) |
| Central Sub-Saharan Africa | -3.01(-3.08--2.93) | -3.54(-4.85--2.21) | -2.44(-2.60--2.28) | -3.84(-5.10--2.56) | -2.67(-2.79--2.56) | -2.84(-3.65--2.03) |
| East Asia | -2.64(-2.75--2.54) | -0.47(-0.56--0.37) | -3.25(-3.44--3.06) | -2.86(-2.95--2.77) | -3.20(-3.29--3.10) | -2.53(-2.58--2.47) |
| Eastern Europe | -0.81(-0.95--0.67) | -0.55(-0.90--0.20) | -5.31(-6.24--4.37) | -2.01(-2.29--1.72) | -4.70(-5.35--4.06) | -1.53(-1.67--1.39) |
| Eastern Sub-Saharan Africa | -3.97(-4.01--3.93) | -4.55(-5.18--3.92) | -2.70(-2.83--2.57) | -5.07(-5.51--4.63) | -2.94(-3.02--2.86) | -5.23(-5.50--4.97) |
| High-income Asia Pacific | -0.17(-0.33--0.01) | 0.41(-0.03-0.86) | -2.90(-3.46--2.35) | -2.44(-2.80--2.07) | -5.21(-5.85--4.56) | -4.17(-4.70--3.63) |
| High-income North America | 0.30(0.17-0.43) | 0.03(-0.38-0.44) | 2.78(1.69-3.88) | -3.38(-4.60--2.15) | -0.03(-1.28-1.24) | -5.05(-5.90--4.19) |
| North Africa and Middle East | -4.65(-4.67--4.62) | 0.15(0.05-0.25) | -4.28(-4.38--4.19) | -1.14(-1.32--0.96) | -4.18(-4.25--4.12) | -1.40(-1.49--1.30) |
| Oceania | -3.27(-3.32--3.22) | 0.23(0.10-0.36) | -2.32(-2.42--2.23) | 0.05(-0.12-0.23) | -2.20(-2.27--2.13) | 0.05(-0.05-0.15) |
| South Asia | -4.68(-4.96--4.40) | -0.48(-0.56--0.40) | -3.45(-3.68--3.23) | -1.41(-1.56--1.27) | -3.21(-3.35--3.07) | -1.24(-1.34--1.15) |
| Southeast Asia | -3.91(-3.98--3.85) | -0.94(-1.02--0.87) | -3.11(-3.15--3.07) | -1.98(-2.04--1.92) | -3.00(-3.06--2.95) | -1.88(-1.94--1.83) |
| Southern Latin America | -3.70(-3.84--3.55) | 0.31(0.10-0.52) | -12.12(-14.08--10.11) | -6.67(-7.75--5.57) | -14.01(-15.39--12.60) | -1.41(-3.04-0.25) |
| Southern Sub-Saharan Africa | -0.19(-0.26--0.12) | -1.64(-1.91--1.38) | 0.66(0.34-0.98) | -2.39(-3.06--1.72) | -0.34(-0.69-0.01) | -3.54(-3.94--3.14) |
| Tropical Latin America | -5.42(-5.64--5.19) | -0.68(-0.97--0.38) | -7.66(-7.84--7.48) | -3.07(-3.44--2.70) | -8.09(-8.24--7.95) | -4.40(-4.80--4.00) |
| Western Europe | 0.01(-0.10-0.13) | 1.43(1.06-1.80) | -4.14(-5.13--3.14) | -8.30(-9.07--7.53) | -8.43(-9.79--7.04) | -6.77(-7.36--6.18) |
| Western Sub-Saharan Africa | -4.60(-4.67--4.52) | -0.35(-1.36-0.67) | -3.49(-3.58--3.39) | -0.35(-1.21-0.52) | -3.54(-3.60--3.48) | -0.00(-0.54-0.54) |
| ASIR:age-standardized incidence rate; ASDR:Age standardised DALY rate; ASMR:Age standardised mortality rate; SDI, socio-demographic index; EAPC, estimated annual percentage change;a EAPC is expressed as 95% CIs. | | | | | | |
