# Supplementary figures and images for: Global trends in typhoid and paratyphoid, and invasive non-typhoidal salmonella, and the burden of antimicrobial resistance: a trend analysis study from 1990 to 2021
Source: Front Med (Lausanne). 2025 May 20;12:1588507. doi: 10.3389/fmed.2025.1588507 (PMC12129787; doi:10.3389/fmed.2025.1588507)

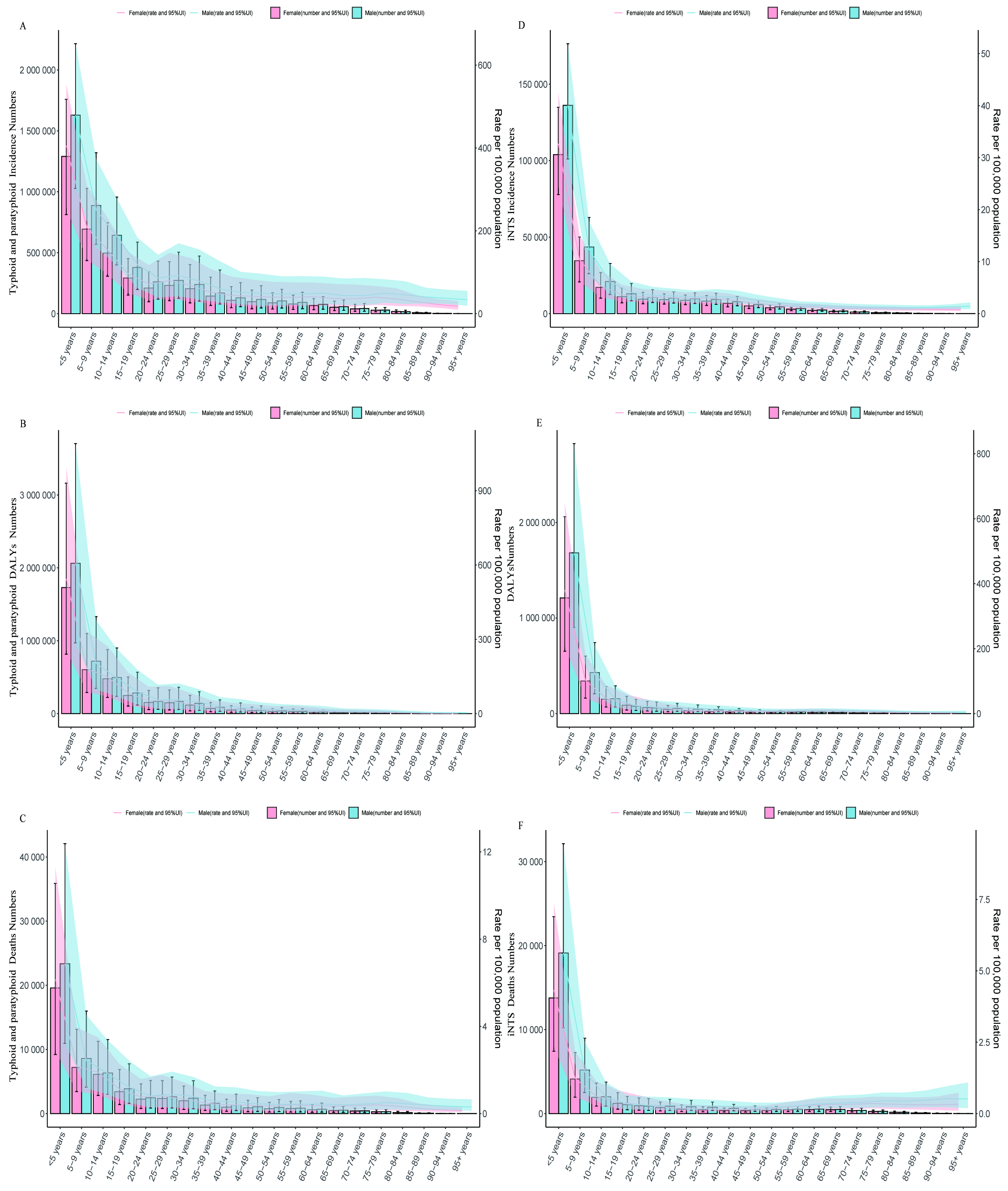

Supplement: Supplementary Figure 1 — Difference in age specific of ASIR, ASDR, and ASMR rates between men and women in 2021. ASIR, age–standardized incidence rate; ASDR, age standardized DALYs rate; ASMR, age standardized mortality rate. [file Image_1.tif]
